# Surgical technique for allogeneic uterus transplantation in macaques

**DOI:** 10.1038/srep35989

**Published:** 2016-10-27

**Authors:** Hideaki Obara, Iori Kisu, Yojiro Kato, Yohei Yamada, Kentaro Matsubara, Katsura Emoto, Masataka Adachi, Yusuke Matoba, Kiyoko Umene, Yuya Nogami, Kouji Banno, Hideaki Tsuchiya, Iori Itagaki, Ikuo Kawamoto, Takahiro Nakagawa, Hirohito Ishigaki, Yasushi Itoh, Kazumasa Ogasawara, Yoko Saiki, Shin-ichi Sato, Kenshi Nakagawa, Takashi Shiina, Daisuke Aoki, Yuko Kitagawa

**Affiliations:** 1 Department of Surgery, Keio University School of Medicine, Tokyo, Japan; 2 Department of Obstetrics and Gynecology, Keio University School of Medicine, Tokyo, Japan; 3 Department of Transplant Surgery, International University of Health and Welfare, Mita Hospital, Tokyo, Japan; 4 Department of Pediatric Surgery, Keio University School of Medicine, Tokyo, Japan; 5 Department of Pathology, Keio University School of Medicine, Tokyo, Japan; 6 Research Center for Animal Life Science, Shiga University of Medical Science, Shiga, Japan; 7 Division of Pathology and Disease Regulation, Department of Pathology, Shiga University of Medical Science, Shiga, Japan; 8 Department of Anesthesiology, Saiseikai Kanagawaken Hospital, Kanagawa, Japan; 9 Safety Research Center, Ina Research Inc., Nagano, Japan; 10 Department of Molecular Life Science, Division of Basic Medical Science and Molecular Medicine, Tokai University School of Medicine, Kanagawa, Japan

## Abstract

No study has reported an animal model of uterus transplantation (UTx) using cynomolgus macaques. We aimed to establish a surgical technique of allogeneic UTx assuming the recovery of a uterus from a deceased donor in cynomolgus macaques. Four allogeneic UTxs were performed in female cynomolgus macaques. Donor surgeries comprised en bloc recovery of organs with iliac vessels on both sides, and/or abdominal aorta/vena cava after sufficient perfusion from one femoral artery or external iliac artery. Before perfusion, 150 mL of whole blood was obtained from the donor for subsequent blood transfusion to the recipient. Four uterine grafts were orthotopically transplanted to recipients. End-to-side anastomosis was performed to the iliac vessels on one side in case 1 and iliac vessels on both sides in case 2; aorto-aorto/cavo-caval anastomosis was performed in cases 3 and 4. Arterial blood flow of the uterine grafts was determined by intraoperative indocyanine green (ICG) angiography. ICG angiography results showed sufficient blood flow to all uterine grafts, and anaemia did not progress. Under appropriate immune suppression, all recipients survived for more than 90 days post-transplantation, without any surgical complications. We describe a surgical technique for allogeneic UTx in cynomolgus macaques.

Uterus transplantation (UTx) has been proposed as a treatment option for women with absolute uterine factor infertility^1^,^2^. Eleven cases of UTx have been reported in Saudi Arabia^3^ in 2000, Turkey^4^,^5^ in 2011, and Sweden^6^,^7^,^8^ in 2012 with either a living donor or a brain-dead donor. Brännström *et al*. described the first human delivery after UTx in Sweden in 2014^7^. To date, five babies have been born after UTx from living donors^9^, whereas there is currently only one case of UTx from a deceased donor^4^. However, UTx from deceased donors should be a priority over UTx from living donors because of the risks for living donors^10^.

Anatomical pelvic structures in cynomolgus macaques are almost identical to those in humans, except that both the superficial and deep uterine veins connect to the external iliac vein in cynomolgus macaques^11^. However, the potential challenge with this species is that delicate surgical techniques for dissection and vascular anastomosis are crucial because of their smaller vessels. We previously established UTx animal models for living-donor surgery^11^ and autotransplantation^12^ using cynomolgus macaques. Moreover, we have been trying to establish models of allotransplantation with living donors by using different surgical procedures from our previous report^13^. However, we encountered many cases of massive intraoperative bleeding and death due to haemorrhage. Therefore, the procurement of wider and longer vessels is needed to establish allotransplantation models in cynomolgus macaques; thus, uterus allotransplantation in this species assuming the recovery of a uterus may be more suitable from a deceased donor than from a living donor.

No study has reported an animal model of UTx assuming the recovery of a uterus from a deceased donor by using cynomolgus macaques. Therefore, we aimed to establish a surgical technique of allogeneic UTx assuming the recovery of a uterus from a deceased donor in cynomolgus macaques.

## Materials and Methods

### Animals

Nine female cynomolgus macaques, *Macaca fascicularis*, (age 5–9 years; average body weight, 3.75 ± 0.86 kg [mean ± standard deviation]) with regular menstrual cycles were used. The animals (n = 9) were either donors or recipients, depending on their blood type and body weight (Table 1). The study was performed in accordance with the recommendations in the Guide for the Care and Use of Laboratory Animals of the National Research Council, and it was approved by the Animal Care and Use Committee of the Research Center for Animal Life Science, Shiga University of Medical Science, Japan (permit number: 2013-4-2).

### Anaesthesia and operative pharmacological treatment

In brief, the animals were anesthetized as previously described^12^. They were intubated and operated on under general anaesthesia with ventilation maintained by isoflurane inhalation (0.5–1.5%; Abbot Japan, Tokyo, Japan). All animals received 25 mg/kg of cefazolin every 3 hours as antibiotic prophylaxis from the initiation of the operation. An anticoagulant (heparin) was administered intravenously 5 minutes before clamping each arterial blood flow. Normothermia and adequate hydration were maintained throughout the operation using heating lights/blankets and a continuous intravenous infusion (10–20 mL/kg/h) of Ringer’s solution.

### Surgical procedures assuming the recovery of a uterus from a deceased donor

**Figure 1:**
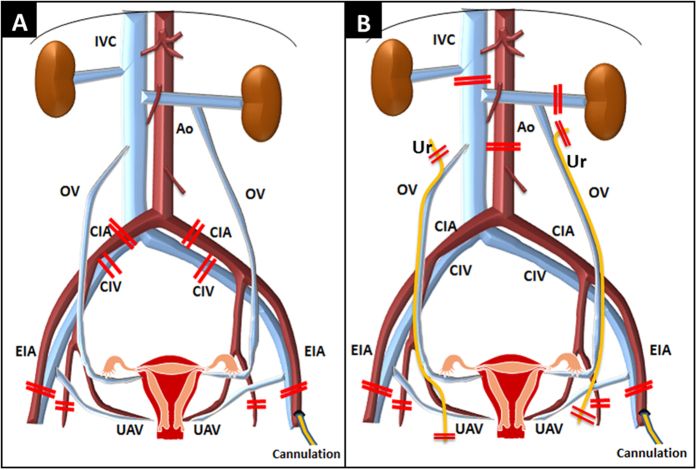
Procurement of the uterus and vessels from the donor. (**A**) Uterus with common iliac arteries and veins on both sides in cases 1 and 2. (**B**) Uterus with the abdominal aorta and inferior vena cava in cases 3 and 4. Ao, abdominal aorta; IVC, inferior vena cava; CIA, common iliac artery; CIV, common iliac vein; EIA, external iliac artery; UAV, uterine artery and vein; OV, ovarian vein; Ur, ureter.

All surgical procedures were performed using sterile techniques and surgical loops. Donor surgeries were performed in accordance with a previous report^14^. For vessel reconstruction, we planned to retrieve the donors’ iliac vessels with the uterine graft in cases 1 and 2, and to retrieve the abdominal aorta and inferior vena cava (IVC) in cases 3 and 4 (Fig. 1).

Before the surgery, a central vein catheter (16 or 18G, Argyle™ CV Catheter Kit, Nippon Covidien Ltd., Tokyo Japan) and an artery line (16 or 18G, Argyle™ CV Catheter Kit, Nippon Covidien Ltd.) were respectively inserted into the left internal or external jugular vein and the left common carotid artery, which were surgically exposed. Thereafter, thoracolaparotomy was performed, and the vessels of the abdominal aorta, vena cava, and those surrounding the uterus were decorticated. Subsequently, the ureters were mobilized from the bladder to the kidneys to enable full access to the uterine and ovarian veins in cases 1 and 2, and then the ureters were immobilized from the uterine arteries, uterine veins, and ovarian veins to prevent vasospasms and injury to the vessels in cases 3 and 4. The vault of the vagina, uterus, and fallopian tubes were completely mobilized with their arterial inflows and venous outflows, including the infrarenal abdominal aorta and IVC (cases 3 and 4), iliac vessels, and ovarian arteries and veins. All iliac vessel branches proximal to the site of the external iliac vessel, which was planned to be resected after perfusion, were ligated and transected with all lumbar branches of the abdominal aorta and IVC, up to the level of aortic bifurcation (cases 1 and 2) or the renal vessels (cases 3 and 4).

After completing all mobilizations, a perfusion catheter (22-G intravenous needle; Terumo Corp., Tokyo, Japan) was inserted into one femoral artery or external iliac artery. Before perfusion, 150 mL of whole blood was withdrawn from the femoral artery or external iliac artery catheter for subsequent blood transfusion to the recipient animal. To maintain the blood pressure of the donor animal, whole blood was withdrawn with rapid intravenous infusion of Ringer’s solution. Immediately after bloodletting, an anticoagulant (heparin, 1000 IU) was administered intravenously. Five minutes later, the abdominal aorta just below the diaphragm was cross-clamped, and the IVC was cut in the pleural space. Simultaneously, the abdominal and pelvic cavities were filled with crushed ice to cool the abdominal organs. Whole organs, including the uterine graft, were perfused with 200–350 mL of cold histidine-tryptophan-ketoglutarate solution (HTK^®^; Custodiol^®^, Nordmedica, Gentofte, Denmark) via the catheter in the femoral or external iliac artery.

Thereafter, the external iliac vessels were tied and transected bilaterally at the distal level where the superficial uterine vein connects to the external iliac vein. The iliac vessels (cases 1 and 2) or abdominal aorta/IVC above the left renal vein (cases 3 and 4) were also transected (Fig. 1A,B). Then en bloc retrieval of the uterus with the adnexa, ovarian vein, and proximal vagina was performed and included the iliac vessels (cases 1 and 2) (Fig. 1A) or infrarenal aorta/IVC (cases 3 and 4) (Fig. 1B). The ovarian veins draining to the renal vein or IVC were left to the uterine graft side (cases 3 and 4). Both the ureters were tied and transected at the distal side from both the renal pelvises in cases 1 and 2, and at the caudal side of the uterine arteries and venous plexus in cases 3 and 4 (Fig. 1B). The ovaries of donors were removed in the operative field. The donors were euthanized by exsanguination, and the uterine graft was preserved in the HTK^®^ solution. Cold ischemia was defined as the time from commencement of cold perfusion in the donor to the removal of the graft from the cold preservation solution. Warm ischemia in the recipient was defined as the time of ischemia during implantation from removal of the uterine graft from the cold preservation solution until reperfusion in the recipient.

### Blood transfusion to the recipient

One hundred fifty millilitre of whole blood, diluted by infusion load, was collected from the donor and stored in an anticoagulant bag including a citrate-phosphate-dextrose solution with adenine (Sepacell Integra CA, Kawasumi Laboratories, Inc., Tokyo, Japan). After leukocytes were depleted using leukoreduction filter sets (Sepacell Integra CA, Kawasumi Laboratories, Inc.), the blood was irradiated (30 Gy) to prevent graft-versus-host disease. This stored donor blood was transfused into each recipient animal during vascular anastomosis and/or after reperfusion of the uterine graft.

### Intraoperative indocyanine green fluorescent angiography

Intraoperative indocyanine green (ICG) was injected intravenously, and the blood supply of organs was monitored at intervals as previously described^12^. The increasing intensity of fluorescence was displayed using the Photodynamic Eye Neo system (Hamamatsu Photonics K.K., Hamamatsu, Japan).

### Recipient surgery

Recipient surgery was initiated about 5 hours after starting the donor surgery to reduce the cold storage time of the uterine graft and shorten the graft waiting time as much as possible. After entering the peritoneal cavity through a midline incision, a total simple hysterectomy was performed, with preservation of the ovaries. The vaginal vault was left open by placing stay sutures on its anterior and posterior wall (eight to 10 interrupted sutures of 3-0 VSORB^®^; Kono Seisakusho, Co., Ltd., Chiba, Japan). Subsequently, iliac vessels on both sides (cases 1 and 2) or the infrarenal abdominal aorta/IVC (cases 3 and 4) was exposed at a distance of approximately 1.5 cm to enable end-to-side anastomosis of the uterine graft vessels.

**Figure 2:**
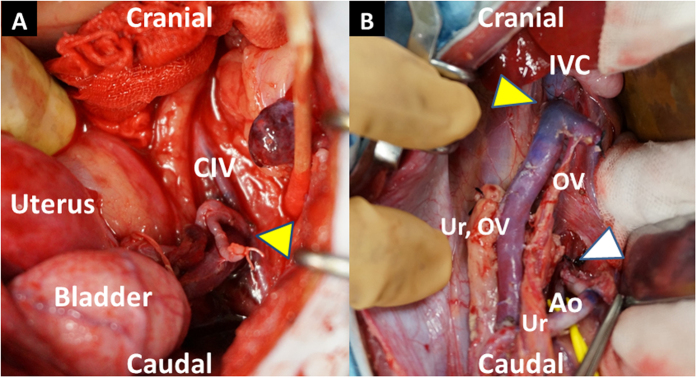
Sites of vascular anastomosis in case 2 (**A**) and case 3 (**B**). (**A**) End-to-side venous anastomosis between the left common iliac vein of the donor and left common iliac vein of the recipient (yellow arrowhead). (**B**) End-to-side venous anastomosis between the inferior vena cava of the donor and inferior vena cava of the recipient (yellow arrowhead), and between the abdominal aorta of the donor and abdominal aorta of the recipient (white arrowhead). CIV, common iliac vein; IVC, inferior vena cava; Ao, abdominal aorta; OV, ovarian vein; Ur, ureter.

The uterine graft was brought into the operative field, and the upper part of the donor vagina attached to the graft was anastomosed with the vaginal vault of the recipient from the posterior wall to the anterior wall by interrupted sutures (3-0 VSORB^®^, Kono Seisakusho, Co., Ltd.) to fix the uterus in the pelvis before performing vascular anastomoses. To keep the graft cold, ice slush was placed around the graft. Before vascular anastomoses, heparin (100 IU) was administered intravenously. After the iliac vein or IVC was cross-clamped, a longitudinal venotomy was made, and end-to-side venous anastomosis was performed using continuous sutures (7-0 or 8-0 Prolene^®^, Johnson & Johnson, Somerville, NJ; Asflex^®^; Kono Seisakusho, Co., Ltd.). Thereafter, the iliac artery or infrarenal abdominal aorta was cross-clamped, and a longitudinal arteriotomy was made. End-to-side arterial anastomosis was also performed using continuous sutures (7-0 or 8-0 Prolene^®^, Johnson & Johnson; 9-0 Asflex^®^, Kono Seisakusho, Co., Ltd.) (Fig. 2). After completing the vessel anastomoses, the vascular clamps were released, and the graft was reperfused. Immediately after reperfusion, a sufficient amount of warm saline was administered into the abdominal cavity. ICG fluorescent angiography was performed after reperfusion. The abdomen was closed with sutures of the fascia and skin.

### Immunosuppressive therapy

As an induction treatment, the animals received antithymocyte globulin (ATG) (Thymoglobulin, Genzyme, Cambridge, MA, USA) intravenously on post-operative day (POD) 0 (day 0 defined as the day of surgery) in cases 1 and 2 and on POD 0 and POD 2 in cases 3 and 4. Maintenance treatment consisted of cyclosporine (CyA) (Sandimmune: Novartis, Basel, Switzerland) in cases 1 and 2 and tacrolimus (TAC) (Prograf; Astellas Pharma, Tokyo, Japan) in cases 3 and 4. The target trough levels for CyA and TAC up to 1 month after the surgery were planned to be within the range of 300–400 ng/mL and 15–20 ng/mL, respectively. As another maintenance treatment, mycophenolate mofetil (MMF) (Cellcept; Chugai Pharmaceutical, Tokyo, Japan) was administered orally in cases 1, 3, and 4. Methylprednisolone (Solu-Medrol; Pfizer, NY, USA) was injected intravenously on POD 0 and was injected intramuscularly daily, starting on POD 1. The dose of methylprednisolone was gradually tapered, and the administration was completed on POD 14 in cases 1 and 2. In cases 3 and 4, 0.5 mg/kg and 0.2 mg/kg of methylprednisolone were administered continuously until the end of observation, respectively.

### Postoperative observation

To monitor for potential rejection after the surgery, the size of the transplanted uterus and blood flow in the transplanted uterine artery were determined by transabdominal ultrasonography under anesthesia. Transvaginal biopsy of the transplanted uterine cervix and body was routinely conducted more than once a month or when considered necessary.

## Results

Nine animals were used in this study (uterine donor, 4; recipients, 5). In case 1, because an animal of a recipient candidate exhibited severe aspiration pneumonia at an early stage of the surgery, another animal was selected as an alternate recipient immediately after euthanizing the first recipient animal.

### Surgical parameters of the donors and retrieval surgeries

The background and surgical details of the four donor animals are shown in Table 2.

With organ perfusion, gross findings in the donor animals showed a change in colour from red to white in all abdominal organs, indicating favourable perfusion of the uterus and all other organs. The duration of the retrieval surgeries (n = 4) averaged 7 hours 23 minutes ± 19 minutes (mean ± standard deviation).

### Surgical parameters of the recipients

**Figure 3:**
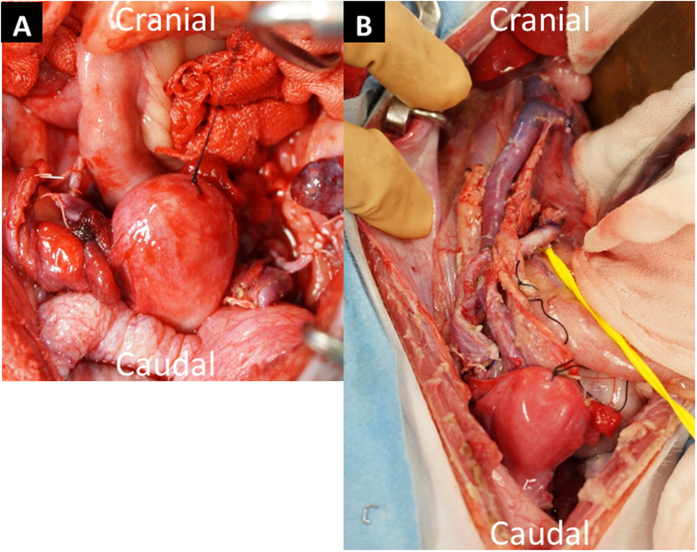
Transplanted uterus in a recipient after reperfusion in case 1 (**A**) and case 3 (**B**). After reperfusion, the colour of the transplanted uterus changed from white to red without uterine congestion.

In the first two transplants, end-to-side anastomoses of the iliac vessels were performed. In case 1 (a substitute recipient was used because the first recipient had a respiratory problem), only anastomosis of the right iliac vessels on one side was performed since there was not enough time to complete both sides because respiratory acidosis rapidly progressed in the new recipient during surgery. In case 2, anastomoses of the iliac vessels on both sides were performed under stable conditions. In cases 3 and 4, end-to-side aorto-aorto and cavo-caval vascular anastomoses were performed (Fig. 3).

**Figure 4:**
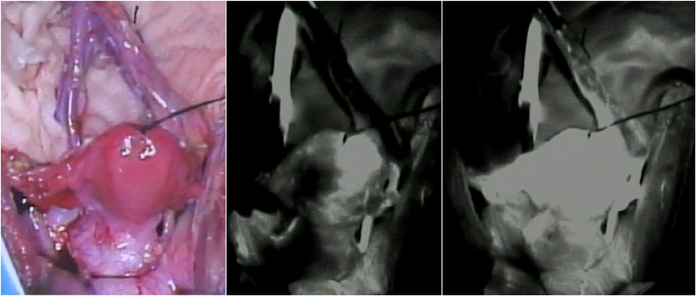
Intraoperative indocyanine green (ICG) fluorescent angiography after reperfusion in case 3. Sufficient blood flow into the transplanted uterus was confirmed.

In all four recipients, good blood flow through the organ occurred immediately after reperfusion, showing a transition in colour in the uterus from whitish to reddish and pulsations through the uterine artery without uterine congestion (Fig. 4). ICG fluorescent angiography performed immediately after reperfusion also showed sufficient blood flow into the whole transplanted uterus (Fig. 4). In case 1, ICG fluorescent angiography results showed rapidly spreading blood flow toward the left from the right side of the uterus, whereas transabdominal duplex Doppler ultrasonography performed after transplant showed satisfactory blood flow in the right uterine artery but not in the left uterine artery. In other recipients, satisfactory arterial flow in both uterine arteries was observed by transabdominal duplex Doppler ultrasonography after surgery.

The cold ischemic time (cases 2, 3, and 4; n = 3) was 45 minutes ± 3 minutes (mean ± standard deviation), and the warm ischemic time (cases 2, 3, and 4; n = 3) was 1 hour 50 minutes ± 12 minutes (mean ± standard deviation). This excluded case 1 because the recipient was replaced with another animal during surgery. The surgical parameters of the recipients are also summarized in Table 2.

### Long-term outcome

**Figure 5:**
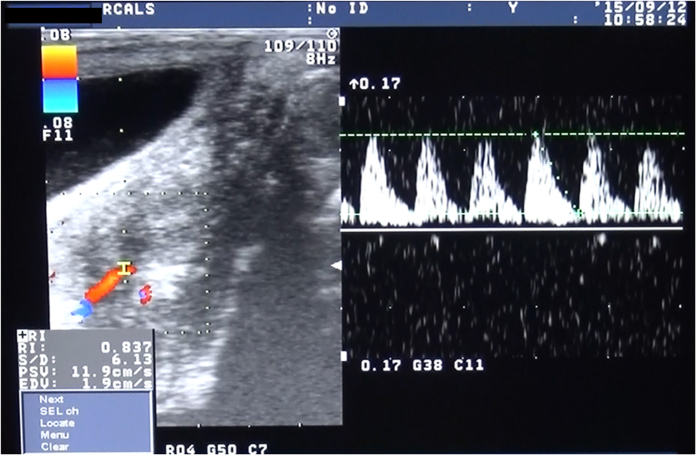
Representative transabdominal ultrasonogram after the transplant. Transabdominal ultrasonography shows good arterial pulsation of right uterine artery 7 weeks after the transplant in case 3.

All four recipient animals survived for more than 3 months after the surgery without any surgical complications. Although rejection was diagnosed in the biopsy samples within 1 month after the surgery in case 4, within 2 months in case 2 and within 3 months in cases 1 and 3, transabdominal ultrasonography showed pulsatile arterial blood flow in the transplanted uterine artery within 1 month after surgery in all cases (Fig. 5). However, none of the animals resumed periodic menstruation after the surgery.

### Blood examination in recipient animals

**Figure 6:**
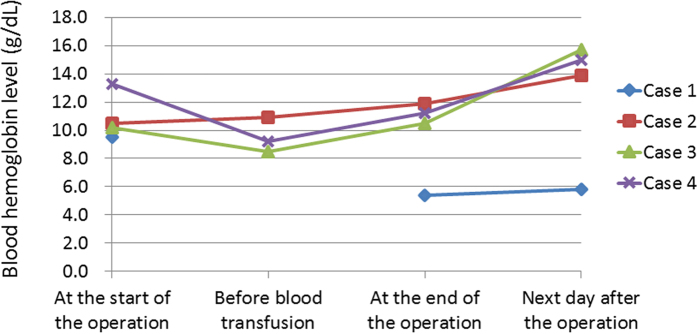
Changes in the blood haemoglobin level (g/dL) during and after the operation in recipients. A decrease in the haemoglobin level was not observed in any cases during the operation with the administration of a blood transfusion, except for case 1, in which heavy bleeding occurred during the surgery. The haemoglobin level was not measured before blood transfusion in the substituted recipient in case 1.

The stored donor blood was transfused into each recipient animal. Changes in the whole blood haemoglobin level in the recipient animals are shown in Fig. 6. In case 1, blood examination of the second recipient animal was not performed before the blood transfusion since the recipient was substituted. A decrease in the blood haemoglobin level was not observed during the operation with the administration of the blood transfusion, except for case 1, in which heavy bleeding occurred during the surgery.

## Discussion

Vascular reconstruction in recipient animals may be easier in deceased-donor models than in living-donor models because much larger vessels could be used for vascular reconstruction in deceased-donor animal models. Recently, we reported a preliminary study of a new perfusion method for UTx with assumed uterus retrieval and preservation from a deceased donor in cynomolgus macaques^14^. In our current experiment, all recipients remained stable during the transplantation procedures, and after reperfusion, the uterine grafts were normal in terms of blood flow and macroscopic appearance, which was confirmed by both ICG fluorescent angiography during the surgery and duplex ultrasound immediately after surgery. Assuming the brain-dead donor model, we performed organ perfusion according to the method we previously reported, which allowed extraction of the uterus while maintaining sufficient perfusion of other abdominal organs^14^. In cases 1 and 2, we used the iliac artery and vein for vascular anastomosis. The general condition of the recipient in case 1 worsened due to aspiration after laparotomy, and because we had to hastily prepare another recipient, the uterus remained in cold storage for an extended period. Moreover, partially because the new recipient had a smaller body size, the iliac vein and artery had an extremely small diameter, which made vascular anastomosis on the left side difficult. There was also not enough time to complete anastomoses on both sides because respiratory acidosis rapidly progressed in the new recipient during surgery. As a result, vascular anastomosis of the transplanted uterus was performed only on the right side (the right iliac artery and vein of the transplanted uterus and the right iliac artery and vein of the new recipient). Nevertheless, because we previously found that it was possible for cynomolgus macaques to become pregnant and give birth even when the uterus had arterial and venous circulation on only one side^15^, we proceeded with vascular anastomosis on only one side. Although circulation to the uterus was maintained after blood flow was restored, there were technical difficulties in case 1. In case 2, we were able to successfully perform anastomosis of the iliac vein and artery on both sides, and blood circulation to the transplanted uterus was extremely good. However, anastomosis of the iliac vein and artery on both sides requires a significant amount of time to complete, which prolongs the warm ischemic time, and in some recipients, the vascular diameter is too small, making it impossible to successfully perform reliable anastomosis. Thus, in cases 3 and 4 we opted to perform vascular anastomosis of the aorta and IVC. As a result, blood loss was reduced because vascular dissection was restricted to the area surrounding the vascular anastomosis of the aorta and IVC. Additionally, because vascular anastomosis was performed on vessels with larger diameters (aorta and IVC of the transplanted uterus and aorta and IVC of the recipient), the surgery was less complicated, and we could effectively reduce the warm ischemic time. However, vascular dissection over a wide area is required to extract the aorta and inferior vena cava en bloc with the uterus from the donor. Thus, this procedure may have the disadvantage of a prolonged operative time in donors.

Although in cases 1 and 2, vascular anastomosis of the ovarian vein of the transplanted uterus was not performed, we were able to establish drainage of venous blood of the uterus through the ovarian vein after UTx in cases 3 and 4 by en bloc extraction of either the left renal vein or IVC, into which the ovarian vein flows. We previously reported that the ovarian vein is an important drainage vein in uterine circulation^16^. Thus, we believe that establishing drainage via the ovarian vein reduced congestion in the transplanted uterus.

There have already been several reports of animal models using anastomosis of the aorta and IVC^17^,^18^,^19^, and they have reported satisfactory outcomes. However, there are no reports of stable surgical procedures of allogeneic UTx using cynomolgus macaques. Our surgical technique is the first example of a successful allogeneic UTx procedure. Another factor that led to the creation of an effective model in this study was the fact that the recipient received an intraoperative blood transfusion. Immediately before donor perfusion, we maintained blood pressure by keeping the donor under transfusion load, removing and storing a large amount of blood, and transfusing this blood into the recipient after irradiation. The blood transfusion could be effective for reducing the recipient’s anaemia and facilitating a stable surgical outcome.

There are a couple limitations in this study. First, we used an animal model; thus, not all parts of the techniques are applicable to clinical settings. Second, factors associated with the long-term outcomes (e.g., immune system suppression and the postoperative organ rejection response) are in progress

## Conclusions

In our study of allogeneic UTx assuming the recovery of a uterus from a deceased donor in cynomolgus macaques, we successfully created a stable allogeneic transplantation technique by performing vascular anastomosis with major blood vessels and administering an intraoperative transfusion of donor blood to the recipient.

## Additional Information

**How to cite this article**: Obara, H. *et al*. Surgical technique for allogeneic uterus transplantation in macaques. *Sci. Rep.* **6**, 35989; doi: 10.1038/srep35989 (2016).

**Publisher’s note:** Springer Nature remains neutral with regard to jurisdictional claims in published maps and institutional affiliations.

## Figures and Tables

**Table 1:** Pre-transplant characteristics of the study subjects.

Case Number	Donor Blood Type	Recipient Blood Type	Donor Age, years	Recipient Age, years	Donor Weight, kg	Recipient Weight, kg
1	AB	AB^*a*^/AB	9	6^*a*^/9	3.86	4.28^*a*^/2.66
2	B	B	6	9	3.37	5.63
3	B	B	9	5	2.78	3.14
4	B	B	9	6	4.17	3.87

No., number. In case 1, an animal as a recipient (^*a*^) was not used because of a respiratory problem at the beginning of surgery. The second animal listed as a recipient in case 1 was a substitute.

**Table 2:** Case summary and surgical data after uterine allotransplantation.

	Case 1	Case 2	Case 3	Case 4
Duration of retrieval surgery	7 h 10 min	7 h 5 min	7 h 23 min	7 h 55 min
Uterine total ischemic time	8 h 41 min	2 h 56 min	2 h 24 min	2 h 25 min
Cold ischemic time	4 h 39 min	49 min	42 min	45 min
Warm ischemic time	4 h 2 min	2 h 7 min	1 h 42 min	1 h 40 min
Time required for vascular anastomosis	1 h 42 min	3 h 12 min	59 min	1 h 10 min
Anastomosed vessels				
Graft (artery/vein)	Right iliac vessels	Both iliac vessels	Ao/IVC	Ao/IVC
Recipient (artery/vein)	Right iliac vessels	Both iliac vessels	Ao/IVC	Ao/IVC
Size of the vascular sutures	8-0, 9-0	8-0	7-0	7-0
Total operation time, recipient	8 h 20 min	9 h 3 min	5 h 47 min	6 h 24 min
Survival status 30 days postoperatively	Alive	Alive	Alive	Alive

Ao, abdominal aorta; IVC, inferior vena cava.
